# Endocrine Disruptors Induced Distinct Expression of Thyroid and Estrogen Receptors in Rat versus Mouse Primary Cerebellar Cell Cultures

**DOI:** 10.3390/brainsci9120359

**Published:** 2019-12-05

**Authors:** Gergely Jocsak, Eniko Ioja, David Sandor Kiss, Istvan Toth, Zoltan Barany, Tibor Bartha, Laszlo V. Frenyo, Attila Zsarnovszky

**Affiliations:** 1 Department of Physiology and Biochemistry, University of Veterinary Medicine, 1078 Budapest, Hungary; kiss.david@univet.hu (D.S.K.); toth.istvan@univet.hu (I.T.); barany.zoltan.balazs@univet.hu (Z.B.); bartha.tibor@univet.hu (T.B.); frenyo.laszlo@univet.hu (L.V.F.); 2 Gedeon Richter Plc., Gyömrői út 19–21, 1103 Budapest, Hungary; iojae@richter.hu; 3 Department of Animal Physiology and Animal Health, Faculty of Agricultural and Environmental Sciences, Szent István University, Páter Károly u. 1, H-2100 Gödöllő, Hungary; zsarnovszky.attila@mkk.szie.hu; 4 Department of Comparative Medicine, Yale University School of Medicine, New Haven, CT 06520, USA

**Keywords:** bisphenol A (BPA), zearalenone (ZEN), arsenic (As), estrogen receptor α (ERα) and estrogen receptor β (ERβ), thyroid receptor α (TRα) and thyroid receptor β (TRβ), primary cerebellar neurons

## Abstract

The endocrine system of animals consists of fine-tuned self-regulating mechanisms that maintain the hormonal and neuronal milieu during tissue development. This complex system can be influenced by endocrine disruptors (ED)—substances that can alter the hormonal regulation even in small concentrations. By now, thousands of substances—either synthesized by the plastic, cosmetic, agricultural, or medical industry or occurring naturally in plants or in polluted groundwater—can act as EDs. Their identification and testing has been a hard-to-solve problem; Recent indications that the ED effects may be species-specific just further complicated the determination of biological ED effects. Here we compare the effects of bisphenol-A, zearalenone, and arsenic (well-known EDs) exerted on mouse and rat neural cell cultures by measuring the differences of the ED-affected neural estrogen- and thyroid receptors. EDs alters the receptor expression in a species-like manner detectable in the magnitude as well as in the nature of biological responses. It is concluded that the interspecies differences (or species specificity) in ED effects should be considered in the future testing of ED effects.

## 1. Introduction

“Endocrine disruptor” (ED) is a now widely used term referring to specific substances that—even in small concentrations—disorganize normal neuroendocrine functions by affecting the physiological regulatory pathways of endogenous hormones. These exogenous substances alter the hormonal balance by dysregulating physiological feedback loops and/or disturbing specific cellular signaling pathways. EDs may have a molecular structure similar to any of the endogenous hormones, thus—as the most common mechanism of action—the substances can bind to their target hormone-receptors, evoke agonistic or antagonistic hormone effects, or block their action [1,2]. Through these receptors, EDs can influence the functions of regulatory centers in the central nervous system (CNS) [3,4], directly in endocrine glands [5] and/or at the effector cells [6].

ED exposure can lead to serious consequences, strongly depending on the developmental stage of the target organism. Life-long complications (modificational effects) can be induced by exposure if it happens prenatally, manifesting in serious developmental alterations. Postnatal incorporation mainly leads to reversible changes in physiological functions (activational effects). Interestingly, ED exposure can be transmitted through the food chain or the placenta and later on through milk. Animal products (e.g., meat, milk) can contain EDs if the source-animal is exposed to them. After consuming the product, EDs will also be “incorporated” into the consumer [7,8,9]. As mentioned above, some EDs can travel through the placenta or through breast milk after birth [10], while in birds, EDs can accumulate in the egg [11].

Misregulation of the neuroendocrine system leads to serious developmental, medical, and even agricultural consequences. EDs have a serious impact on the physiology of the organism, depending on the targeted cells, tissues or organs. Not only the functions of neuroendocrine organs [12,13] and certain processes in the central nervous system [14] can be disrupted, but by altering the central regulation of the body, EDs also impair liver and kidney homeostasis and their functions during detoxification; cellular components of the blood are also influenced by ED exposure; the immune response is affected by changing the quality and quantity of immune cells [15] and contributes in the manifestation of several metabolic diseases [16]. Furthermore, EDs strongly interfere with the reproductive physiology of animals (as their “main target”), thus causing major economic losses through lowering the productivity of the livestock [15,17], not to mention the possibility of similar effects in humans.

There are several testing methods concerning the identification of EDs; however, these approaches mainly act as guidance. The risk evaluation of EDs is impacted by modes of action, appropriate dose, and exposure from the target substance. The number of validated test systems for EDs are insufficient to accurately identify endocrine disruption [18]. Aside from the toxicity of individual compounds, effects at low concentrations, delayed effects, environmental factors of the experiments, and the life stages and sensitivity of the model species need to be sufficiently reliable during risk assessment [19,20]. Furthermore, endocrine-mediated (the effect is a consequence of a specific chemical interaction with a receptor or other molecular component of the endocrine system) and non-endocrine mediated (adverse effects occur by other mechanisms) adverse effects should be distinguished by these methods [21].

To identify a molecule as an ED, the substance has to have the ability to cause a change in endocrine functions. The majority of used tests do not meet these criteria [22]. The assessment strategy of the European Food Safety Authority (EFSA) involves four groups for its evaluation tests: in vivo, in vitro, “EATS (estrogen, androgen, thyroid, and steroidogenic modality) mediated” and “Sensitive to, but not diagnostic of, EATS” categories. Based on a complex evaluation system, all the ED criteria—endocrine activity, adversity and the occurrence of a biological link between the two—can be tested in the case of the investigated substances. Although some non-animal based options are available (e.g., yeast strains with appropriate nuclear receptors acting as in vitro bioassays [23]), the former two test types are mainly conducted on animal models [24,25].

Classical toxicology focuses on “endpoints” of chemical action that are different than in the case of ED research; in the latter case, the effect of substances may manifest slower, causing long term hormonal, developmental, and behavioral changes [26]. Several of these alterations show species-specificity, predominantly exerted on the reproductive organs of animals or humans [26,27,28,29]. The physiological background of these differences is not known, however, according to Benigni et al. [30], the basis of disparity can be found on the level of nuclear receptors and their interaction with EDs.

EDs can connect to thyroid- (TR) and estrogen receptors (ER), resulting in a potency of endocrine disruption. Available data suggest that estrogens—estrone (E1), 17β-estradiol (E2), and estriol (E3)—and thyroid hormones (THs)—triiodothyronine (T3) and thyroxine (T4)—are equally important regulators of CNS development [31,32]. In the developing cerebellum and possibly in any other brain area, a complex, interconnected effect can be seen between the respective hormones of TRs and ERs, maintaining the physiological levels of these specified receptors [33,34].

Based on our studies and the existing literature, a natural balance between estrogen- and thyroid hormones can be disturbed by ED exposure, leading to complex, far-reaching consequences in the targeted organs. These effects are possibly more pervasive than currently believed [34,35,36]. 

Preliminary studies in our laboratory suggested that there might be interspecies differences in the reaction of experimental animals to ED exposure. Therefore, in the present study, we hypothesize that some EDs may have distinct effects on the estrogen- and thyroid system in rats and mice. To test this hypothesis, a generally accepted and widely used in vitro model, the primary cerebellar cell culture derived from either rat or mouse pups, was used [37,38,39,40]. 

The neural development is regulated through ERs and TRs (and their respective hormones, E2 and THs) in the cerebellum and other brain areas [37,41,42]. ERs are expressed in the neural tissue from the postnatal period in rats; the receptor expression is developmentally regulated [43]. During postnatal life, ERβ receptors are expressed in many types of cerebellar cells: on Purkinje cells, stellar cells, basket cells, and Golgi cells. The migrating glial cells and the developing neurites also express the ERβ receptor protein [37,44]. Likewise, TRs are found in every cell of the cerebellum; thus, THs affect the development of this brain area through their cognate receptors [45,46]. Altering the delicate balance between estrogen- and thyroid regulation—either through the changes of receptor expression or through altered receptor function—results in developmental deviations of the cerebellum. Animal cells respond to external signals by modulating their receptor composition (quantitatively and qualitatively); therefore, a change can be observed in the receptor mRNA levels after an ED exposure. In addition to single endocrine disruptor treatment—either bisphenol-A (BPA), zearalenone (ZEN), or arsenic (As)—ED and native hormone co-treatment effects were also investigated. 

Bisphenol-A (BPA) is a well-known and widely researched organic compound synthesized by the plastic industry. The molecule can be found in numerous plastic containers—in water or feeding bottles, food containers, sports equipment, re-usable plastic tableware, CDs, and DVDs—due to its positive effect on the quality of manufactured plastic. BPA is also present in dental sealants and other medical devices [47]. Epoxy resins in water pipes or the plastic layer inside canned food containers can possibly contain BPA, from which potential contamination of the food is very likely. BPA is also present in thermal paper such as that used as sales receipts. 

Zearalenone, also known as F-2 mycotoxin, is an exceptionally strong mycoestrogen, a mixed agonist-antagonist of specific receptors (e.g., ERα and ERβ) [48]. The toxin is synthesized as a secondary metabolite by Fusarium and Gibberella molds. The fungal infection of agricultural grain products (for example, maize, barley, oats, wheat, rice, and sorghum) may start at the cultivation area, before or after harvest (in the silage). Improper storage favors the proliferation of the fungi so that they may reproduce in a short time on the forage kept in a dark and damp environment [49]. A study in the EU examined 5010 grain samples in 2004 and found F-2 mycotoxin contamination in 32% of samples [50]. In Hungary, Fazekas et al. [51] found ZEN in 17% of the analyzed probes. These studies prove that ZEN exposure is a serious problem in agriculture, implying a notable risk on animals and humans as well. 

As III (arsenite; +3) and As V (arsenate; +5)—naturally occurring oxidation states of As—can be found in minerals (in combination with sulphur and metals) or in pure form as a crystal; however, it may naturally appear in the groundwater as a contaminant of drinking water globally. The accumulation of As affects millions of people around the world (even in first world countries such as Denmark). Originating from the industrial use of As in semiconductors, in alloys of lead (ammunition, car batteries), and improper waste disposal augmented by the agricultural use (as a component in herbicides, pesticides, and insecticides) and a wood preservative agent, As pollution continuously increases with the growing anthropogenic activity [52,53].

Hypothesis:

This study reports the first results of a larger and complex series of experiments in which we test the effects of BPA, Zea, and As on estrogen receptor and thyroid receptor mRNA and protein expression in a rat and mouse model. In order to elucidate the putative endocrine-disrupting effects of the above chemicals, rat and mouse cerebellar granule cell cultures were drawn into investigations. EDs and native hormones (E2, TH, in physiologic concentrations) were applied to test the following hypotheses:

1. Significant differences will be detected between rat and mouse ER and TR mRNA expression levels.
2. Differences in ED effects between species will be observed in magnitude only, while the inhibitory or stimulatory nature of the effects on hormone receptor expression remains similar in the compared species.

## 2. Materials and Methods 

All of the experiments were conducted by the method previously described in Jocsak et al. (2016) [35], with additional modifications.

### 2.1. Reagents and Materials

Culture media (Dulbecco’s modification of Eagle’s medium [DMEM], Ham’s F-12 50/50 mix), and TRI reagent were from Invitrogen (Carlsbad, CA, USA). Penicillin/streptomycin and heat-inactivated fetal bovine serum were purchased from GIBCO (Budapest, Hungary). Cytosine β-D-arabinofuranoside (CAS: 147-94-4), 17β-estradiol (MDL number MFCD00133134; CAS: 50-28-2), 3,3′,5-triiodo-l-thyronine (purity: 96%; CAS: 6893-02-3), zearalenone (purity: ≥99%; CAS: 17924-92-4), bisphenol A (purity: ≥99%; CAS: 80-05-7), and sodium (meta)arsenite (purity: ≥90%; CAS: 7784-46-5) were purchased from Sigma-Aldrich (Budapest, Hungary). Bicinchoninic acid (BCA) kit from Pierce (Rockford, IL 61111, USA). 

### 2.2. Animals

Sprague–Dawley rats were purchased from TOXI-COOP Zrt. Budapest, Hungary, C56BL/6 mice were purchased from HAS Biological Research Centre, Szeged, Hungary, and maintained in the animal facility of the University of Veterinary Medicine, Budapest. Animals were kept under standard laboratory conditions, allowed free access to food and water, and maintained on a 12/12-h light/dark cycle until the time of sacrifice. The animals were treated according to the EC Council Directive of 24 November 1986 (86/89/EEC) and all procedures were reviewed and approved by the local ethical committee (Animal Welfare Board at University of Veterinary Medicine and regional animal welfare authority, ID: PE/EA/1252-6/2016, date: 05/2016, Pest Megyei Kormányhivatal).

### 2.3. Preparation and Culture of Cerebellar Granule Cells 

Dissociated cerebellar cells were prepared from seven-to-nine day-old Sprague-Dawley rats and six-day-old C56BL/6 mice, from both sexes, since no gender differences were observed in the present and previous studies. Neuronal cultures were obtained by the method previously described, with small modifications [54]. The removed cerebella were dissociated without enzymatic treatment by repeated trituration. Triturated cells were filtered through a nylon cell strainer by gravity (pore size 70 and 40 µm) to remove large, non-dissociated cell clumps and non-neuronal cells. Cells were seeded in poly-L-lysine pre-coated Petri dishes at densities of 2300–2700 cells/mm^2^ and maintained in culture for 5–7 days (37 °C, 5% CO^2^) in serum and steroid free Dulbecco’s modified Eagle medium supplemented with 5 µg/ml insulin, 5 µg/ml transferrin, 5 µg/ml selenium, and 20 mM KCl for mild depolarization and survival of the cells [55,56,57]. To inhibit the proliferation of non-neuronal cells, 10 μM cytosine β-D-arabinofuranoside (Ara-C; Sigma Aldrich Ltd., Hungary) was added 24 h after seeding. The resulting cultures consisted of non-clustered, granule cell type neurons (~ 95%).

### 2.4. Treatments

Cells were treated after five (mouse-derived) or seven days (rat-derived) grown in culture with the following hormones, at physiologically relevant concentrations: 1.16 × 10^−10^ M 17β-estradiol (E2), 9.2 × 10^−10^ M 3,3’,5-triiodo-L-thyronine (T3), and/or the endocrine disruptors: zearalenone (ZEN) 10^−10^ M, bisphenol A (BPA) 10^−10^ M, and arsenic 2.5 × 10^−7^ M. Treatments were applied for six hours before cell harvesting. Quantity of hormones and EDs were chosen according to the sensitivity of the applied neuronal cell culture system and to our previous experiments where the most effective dosage in proving ED effects was determined [58,59]. In the case of DMSO vehicle, it was used at 0.1% concentration and had no effect on cultured cells. Experiments were repeated at least 5 times. 

### 2.5. Revers Transcription- and Quantitative-RT-PCR

Total RNAs were extracted from mouse and rat primary cerebellar cultures using TRI reagent following the manufacturer’s protocol (Invitrogen, Carlsbad, CA, USA) and was purified from samples with the direct-zol RNA miniprep kit (Zymo Research, Irvine, CA 92614, USA). RNA integrity was electrophoretically verified by ethidium bromide staining and by OD260/OD280 nm absorption ratio >1.85. Three μg of total RNA was reverse transcribed by RT-PCR in a final volume of 30 μL using M-MLV reverse transcriptase (Promega Corporation, Wisconsin, USA) and oligo(dt) primers. Subsequently, 2 µL of the resulting cDNA samples were analyzed in triplicates by qRT-PCR (Master SYBRGreen, Hoffmann-La Roche, Basel, Switzerland) in a LightCycler 2.0 F. device (Hoffmann-La Roche, Basel, Switzerland) using glyceraldehyde 3-phosphate dehydrogenase (Gapdh) and/or Beta-actin as an endogenous control for normalization of the data. Primer pairs were designed using NCBI’s primer designer Primer-BLAST, or were taken from literature, and used at 0.2 mM concentration. The primer sequences used for mouse and rat Gapdh, beta-actin, ERα, ERβ, TRα, and TRβ are given in Table 1. The primer sequences used for amplification of rat TRα and TRβ were as previously published by Billon [60] and Kariv [61], respectively. Rat ERα and ERβ primer sequences were as published by Vaillant et al. [62]. The LightCycler PCR thermal cycling was accomplished by a two-step temperature protocol with a 95 °C for 10 min for enzyme activation followed by a 4-segment amplification and quantification program. The real-time PCR threshold cycle (Ct) data were analyzed using the REST-XL software version 2.0 (GenEx—BioMcc, TUM, München, Germany) [63]. Cycle threshold values were normalized to those of Gaphd or beta-actin. The relative expression ratios of mRNA (fold changes) were calculated using the 2^−ΔΔCt^ method.

### 2.6. Data Analysis

All data that have been presented are representative of at least three independent measurements. Statistical analyses were conducted using Excel (Microsoft, Microsoft Co., Redmond, WA, USA) and GraphPad Prism version 4 (GraphPad Software, San Diego, CA, USA) by means of two-way ANOVA with Bonferroni post-tests and/or unpaired *t*-tests. Statistical analyses were carried out by the Department of Biomathematics, University of Veterinary Sciences, Budapest, Hungary.

## 3. Results

### 3.1. Expression of Estrogen Receptor Alpha (ERα)

**Figure 1:**
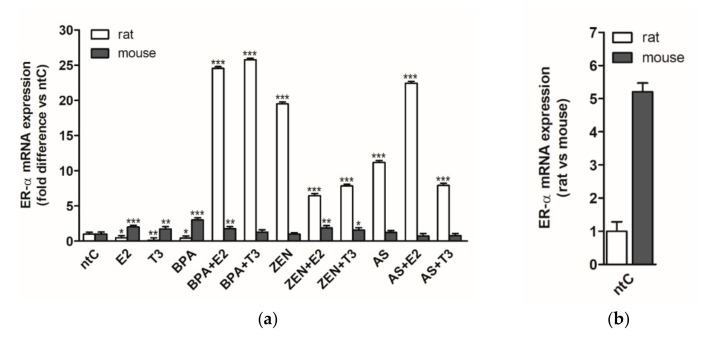
ERα mRNA expression in rat and mouse cerebellar granule cells treated with 17β-estradiol (E2), triiodo-thyronine (T3), bisphenol A (BPA), zearalenone (ZEN), arsenic (As), alone or in combination with the hormones. (**a**) Relative expression level of the ERα gene was analyzed by qRT-PCR and normalized to the average of the control gene β-actin or Gapdh. Shown *p*-values were calculated compared to ntC: (*) *p* < 0.05, (**) *p* < 0.01, (***) *p* < 0.001. (**b**) Relative expression of ERα mRNA in rat versus mouse non-treated controls, normalized to β-actin or Gapdh (*p*-value not shown). The data shown here are the mean ± standard deviation (SD) of at least three independent experiments (*n* = 5 per treatment).

ERα gene transcription was quantified following 6 h treatment with the different hormones and endocrine disruptors, alone or in combination with these hormones in both rat and mouse neuronal cultures (Figure 1). In rat cerebellar granule cells, both hormones, E2 and T3, had a negative effect on transcription of ERα receptor (*p* < 0.05 and *p* < 0.01, respectively). In a similar fashion, BPA also decreased significantly the expression of ERα receptor (*p* < 0.01); however, in the presence of E2 and T3 hormones, BPA increased highly the ERα mRNA levels (*p* < 0.001). ZEN applied alone increased the mRNA levels of ERα compared to non-treated control (ntC), *p* < 0.001, whereas the presence of both hormones significantly decreased the ERα expression compared to ZEN (*p* < 0.001). The arsenic effect on ERα gene transcription was less pronounced than that exerted by ZEN, yet was significantly high compared to non-treated controls and hormones (*p* < 0.001). E2 and T3 modulated in a reverse manner the As induced gene transcription; E2 increasing and T3 decreasing the transcriptional rates in response to As (*p* < 0.001).

In mouse cerebellar granule cell cultures, both hormones increased the ERα expression compared to non-treated controls (*p* < 0.001 and *p* < 0.01, respectively), and compared to those observed in rat neuronal cultures. BPA also significantly increased the levels of ERα mRNA expression, while the presence of E2 and T3 changed the transcriptional profile leading to significantly decreased expression rates (*p* < 0.001) compared to BPA. In mouse neurons, ZEN-induced ERα levels did not alter significantly from those detected in the ntC, whereas the presence of E2 and T3, both led to increased mRNA levels (*p* < 0.01) following combined ZEN treatment. A different expressional pattern could be observed in the case of As, where the expression of ERα was not altered by As alone or in combination with E2 and T3.

Comparing the two neuronal cultures, the effects of hormones and endocrine disruptors on ERα transcription differed significantly. High induction of receptor transcription could be observed by endocrine disruptors in rat neurons contrasting the mRNA levels observed in mouse cultures, where the evoked expression levels showed a higher deviation within the different EDs applied. In mouse neurons, only BPA increased the ERα expression significantly compared to the other EDs and ntC, while modulation of receptor expression in the presence of hormones was detected in the case of BPA + E2, ZEN + E2, and Zen + T3. Interestingly, in rat neurons, the BPA alone had a negative effect on ERα mRNA levels, decreasing the expression significantly (*p* < 0.05). Treatments with E2 and T3 hormones alone resulted in opposing read-outs within the two experimental models, with high estrogen and thyroid receptor sensitivity in mouse-derived neurons and decreased ERα receptor expression in rat neuronal cultures.

### 3.2. Expression of Estrogen Receptor Beta (ERβ)

**Figure 2:**
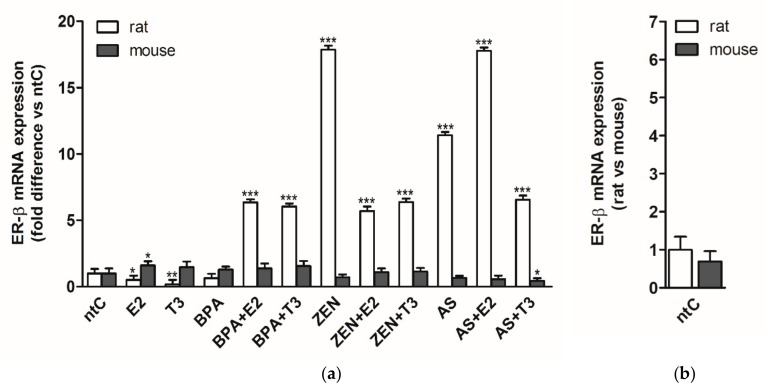
ERβ mRNA expression in rat and mouse cerebellar granule cells treated with 17β-estradiol (E2), triiodo-thyronine (T3), bisphenol A (BPA), zearalenone (ZEN), arsenic (As), alone or in combination with the hormones. (**a**) Relative expression level of the Erβ gene was analyzed by qRT-PCR and normalized to the average of the control gene β-actin or Gapdh. Shown *p*-values were calculated compared to ntC. (**b**) Relative expression of Erβ mRNA in rat versus mouse non-treated controls, normalized to β-actin or Gapdh (*p*-value not shown). The data shown here are the mean ± standard deviation (SD) of at least three independent experiments (n = 5 per treatment).

In rat neuronal cultures, the ERβ expression patterns were, at some extent, similar to those observed in the case of ERα receptor in the same experimental model, with some differences in mRNA levels (Figure 2). BPA administered alone did not cause change in the ERβ expression, while the E2 and T3 hormones alone had a negative effect on receptor expression as observed for ERα in rat neurons. Both E2 and T3 increased the ERβ mRNA levels when co-administered with BPA (*p* < 0.001), whereas in the case of ZEN, the presence of hormones significantly decreased the ZEN induced ERβ expression (*p* < 0.001) compared to ZEN. ZEN alone induced the highest expression rates of ERβ within the treatment groups. For ERβ expression induced by arsenic (*p* < 0.001) compared to ntC), the presence of E2 had a cumulative effect on ERβ transcription, while T3 regulated the receptor expression negatively (*p* < 0.001). 

In mouse neuronal cultures, few changes were detected in the ERβ receptor expression which remained mostly unchanged by the different treatments. Only E2 increased the mRNA levels significantly (*p* < 0.05) and the As + T3 combination caused a significant decrease in receptor expression (*p* < 0.05). 

The two neuronal cultures show a very different ERβ receptor sensitivity to the applied hormones and endocrine disruptors, the mouse primary neuronal cultures proving to be less or non-responsive in response to hormones and EDs.

### 3.3. Expression of Thyroid Hormone Receptor Alpha (TRα)

**Figure 3:**
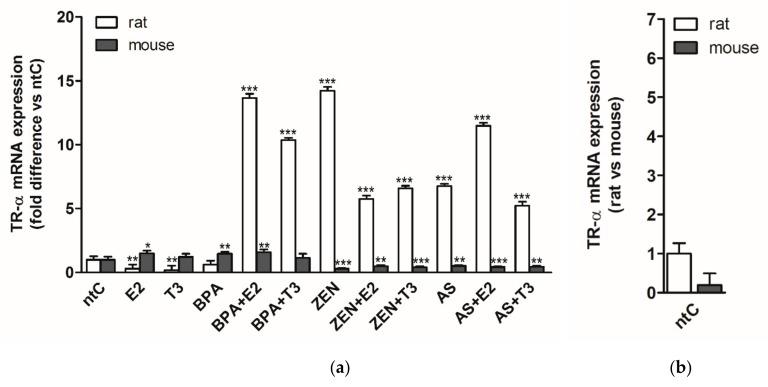
TRα mRNA expression in rat and mouse cerebellar granule cells treated with 17β-estradiol (E2), triiodo-thyronine (T3), bisphenol A (BPA), zearalenone (ZEN), arsenic (As), alone or in combination with the hormones. (**a**) Relative expression level of the TRα gene was analyzed by qRT-PCR and normalized to the average of the control gene β-actin or Gapdh. Shown *p*-values were calculated compared to ntC. (**b**) Relative expression of TRα mRNA in rat versus mouse non-treated controls, normalized to β-actin or Gapdh (*p*-value not shown). The data shown here are the mean ± standard deviation (SD) of at least three independent experiments (*n* = 5 per treatment).

TRα gene transcription was quantified after 6 h treatment with the different hormones and endocrine disruptors, alone or in combination with these hormones in both rat and mouse neuronal cultures (Figure 3). 

In rat cerebellar granule cells both hormones, E2 and T3 led to a significant drop in TRα expression compared to ntC (*p* < 0.01 for both). BPA did not alter the TRα expression levels, while both ZEN and As highly increased the receptor expression (*p* < 0.001). When applied with the hormones, BPA significantly increased the mRNA levels compared to BPA and ntC. In the case of ZEN, both hormones affected negatively the expression levels elicited by ZEN treatment. In combination with the As treatment, E2 induced the transcription of TRα, while T3 negatively modulated the As-induced expression patterns of TRα. Except for BPA, all endocrine disruptors, applied alone or with hormones, significantly increased the expression of TRα compared to ntC (*p* < 0.001). 

In mouse neuronal cultures, an opposite effect could be observed compared to rat neuronal cultures following hormonal treatment where E2 increased the TRα expression; however, T3 had no major effect on receptor expression. Moreover, BPA alone also led to increased mRNA levels compared to ntC (*p* < 0.01) and to those observed in rat neurons. Concurrent administration of BPA with hormones did not result in TRα expression differences. Hormones elicited a modulatory effect on ZEN induced negative TRα expression levels (*p* < 0.001); both E2 and T3 increasing the mRNA levels compared to sole ZEN treatment (*p* < 0.01 and *p* < 0.05, respectively) Similarly to ZEN, As had a negative effect on TRα expression, significantly decreasing the mRNA levels (*p* < 0.001). When applied with hormones, E2 decreased further the low mRNA levels induced by As alone.

Comparing the two neuronal cultures, an opposite response in mRNA levels to the different hormones and EDs could be detected, the rat neuronal cultures exhibiting a high, ligand selective expression of TRα to Zen and As in the presence or absence of hormones, whereas a high repression of transcriptional machinery is observed in mouse neuronal cultures to these treatments. 

### 3.4. Expression of Thyroid Hormone Receptor Beta (TRβ)

**Figure 4:**
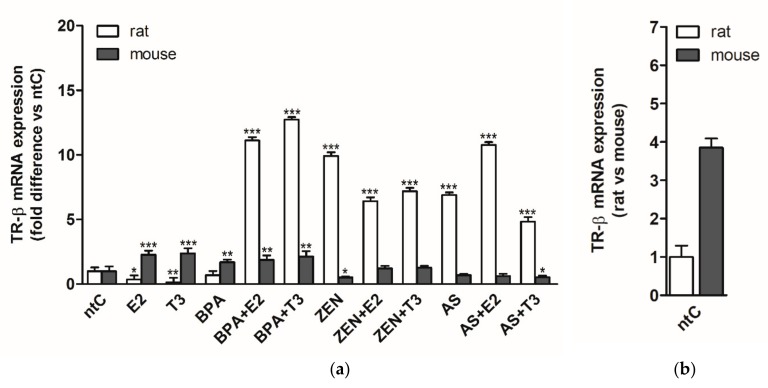
TRβ mRNA expression in rat and mouse cerebellar granule cells treated with 17β-estradiol (E2), triiodo-thyronine (T3), bisphenol A (BPA), zearalenone (ZEN), arsenic (As), alone or in combination with the hormones. (**a**) Relative expression level of the TRβ gene was analyzed by qRT-PCR and normalized to the average of the control gene β-actin or Gapdh. Shown *p*-values were calculated compared to ntC. (**b**) Relative expression of TRβ mRNA in rat versus mouse non-treated controls, normalized to β-actin or Gapdh (*p*-value not shown). The data shown here are the mean ± standard deviation (SD) of at least three independent experiments (*n* = 5 per treatment).

In rat neuronal cultures, the TRβ expression showed similar patterns to those observed for TRα. Both E2 and T3 hormones applied alone decreased the TRβ expression (*p* < 0.05 and *p* < 0.01, respectively) compared to ntC (Figure 4). BPA alone did not alter the receptor expression; however, when applied with E2 and T3, a high increase of TRβ mRNA levels were detected compared to BPA or to non-treated controls (*p* < 0.001), T3 inducing higher mRNA levels compared to E2. Treatment groups with ZEN and/or hormones resulted in comparable expression patterns as those observed in the case of TRα with lower ZEN induced mRNA levels. As and As with hormones induced TRβ expression with comparable levels to those observed in the case of TRα. 

In mouse neurons, the expression of TRβ in response to the different treatments was more divergent compared to those observed in the case of TRα. Both hormones increased the expression of TRβ significantly (*p* < 0.001) compared to non-treated controls. TRβ expression level induced by BPA was similar to those observed when BPA was co-administered with E2 and T3, compared to ntC (*p* < 0.01). ZEN applied alone decreased the receptor expression (*p* < 0.01), whereas the presence of hormones reverted the negative effects exerted by ZEN resulting in basal mRNA levels comparable to ntC. Arsenic had no significant effect on TRβ expression. When applied with hormones, E2 did not impact the receptor expression, while T3 increased the inhibitory effects of As on TRβ expression (*p* < 0.05).

A reverse expression pattern of TRβ in response to E2 and T3 hormones was observed between the two rat and mouse neuronal cultures. BPA increased the expression of TRβ in mouse neurons, but BPA had no effect on TRβ expression in rat neuronal cultures. ZEN exerted an opposite effect on receptor expression in the two cultures, increasing the expression in rat neurons and modulating the transcription in a negative fashion in mouse cultures. Rat neuronal cultures proved to be highly sensitive to EDs applied alone or in combination with hormones except for BPA and compared to mouse neurons where the sensitivity to EDs was less pronounced, albeit a high modulatory effect of E2 and T3 on receptor expression was found.

## 4. Discussion

### 4.1. ED Exposure and Effect in Animals

The main route of BPA exposure is ingestion (as a food contaminant), but it also absorbs through the skin. In the body, BPA is able to mimic the natural hormone E2 [64]; therefore, since 1997, it has been considered as an ED. Firstly, it was considered as a weak estrogen-mimicking substance with minimal health risk, but later studies on the molecular mechanisms revealed that even in small concentrations (between 0.2–0.002 ng/mL), receptor binding (to ERα and ERβ) activates and/or stimulates a huge variety of different intracellular pathways and cell responses [65,66,67]. The safe dose of daily BPA intake per day is below 50 μg/kgbw according to the Environmental Protection Agency (EPA) [67]. Recent reports suggest that BPA exposure above the safe dose can be linked to several reproductive disorders: in Long Evans rats (and in additional unspecified rat strains) 50 μg/kgbw BPA exposure resulted in reduced fetal survival, advancement of puberty, reproductive development, ovarian malformations, and reductions in maternal and fetal body weight [68,69]. Besides the negative impact on reproductive functions, other adverse effects have also been indicated. For example, the quality and quantity of the immune response (the T cell count, B cell functions, and dendritic cell and macrophage physiology) are altered by the disruptor as well [70]. Based on the diverse mechanisms of endogenous estrogen, the estrogen-mimicking BPA might be responsible for a huge number of other pathological conditions. Furthermore, studies indicated that BPA (between 10 µM to 100 nM) also affects the thyroid functions of the organism by binding to thyroid hormone receptors (unspecified TR in an unspecified rat strain; TRα and TRβ in human TSA201 cells) [71,72]. Normal thyroid and estrogen signaling in the brain is essential for healthy CNS development in infants. Therefore, the manifestation of serious developmental disorders in neonates is likely if pregnant women are exposed to BPA during gestation [73,74]. BPA is poorly soluble in water, but it dissolves in organic solvents. Derived from consumer products, it can significantly contribute to the pool of estrogenic substances in the environment [75].

Zearalenone can be transferred into the bloodstream of livestock (or humans) by the ingestion of contaminated grain products (as forage or processed food). In some of the internal organs (mostly liver, kidneys, testes, prostate, ovaries, intestines, and the hypothalamus), a specific enzyme group called 3α- and 3β- hydroxysteroid dehydrogenase (HSD) break ZEN down into metabolites (α- and β-zearalenol, zearalanon, α- and β-zearalenon). Some of the metabolites possess an even stronger estrogen-like effect than the original molecule (for example, the estrogen-like effect of α-zearalenol is 3–100 times higher than ZEN) [76,77]. After entering the circulation, ZEN and its metabolites target the ERs all over the body. The chemical bond between the ER and the substances is 20 times weaker than in case of E2 [48]. These mycotoxins have an estrogen-like molecular structure, which allows them to exert an anabolic effect on the target cells. Neuroendocrine and reproductive organs are the primary target of ZEN; here the mycotoxin causes severe anatomical and physiological disorders leading to anoestrus, pseudopregnancy, elevated chance of stillbirth, and developmental disorders in the developing fetus of the pregnant animal. The effect of ZEN is not restricted on reproduction; however, it affects the immune system, the physiology of the bones, liver, kidneys, and the CNS as well [15,17,48,49,78,79]. The mycotoxin (and its metabolites) are able to penetrate the blood–brain barrier (BBB); therefore, they easily reach the cells of the CNS [14]. Furthermore, in Sprague–Dawley (tested ZEN dosage between 0.02–1 mg/kgbw) rats and in Swiss albino mice (1.5–6 mg/kgbw), the mycotoxin may also disintegrate the BBB, possibly weakening the animal against other toxins and diseases [80,81,82]. After crossing the “protective guard” of the CNS, as a result of its E2-like structure, ZEN is able to directly affect the physiology of the cells in the CNS, due to the abundance of ERs in neurons and glial cells [83].

Arsenic has the potency to affect the neuroendocrine system. As III can connect to ERs, thus changing expression levels of E2 responsive genes [84,85,86]. As V acts indirectly on the endocrine systems after it is enzymatically converted to As III, acting as an arsenite “reservoir” [87]. Due to its estrogenous activity, Arsenic is associated with different cancer types: it has been indicated to provoke cancer in skin, lung, breast, and bladder, and is also involved in cardiovascular diseases; by being a potent ED in humans, it disrupts gonadal, adrenal, and thyroid functions as well [88,89,90]. The role of arsenic has been indicated in many reproductive disorders [91,92]. Besides the estrogenic effects, As III affects the thyroid functions; in amphibian models (*Silurana tropicalis*), it lowers the TRβ and deiodinase type 2 mRNA levels [93].

### 4.2. Differences in mRNA Expression between Species

As a response to an environmental change, differences in mRNA expression between species have already been observed in several experiments; therefore, conclusions drawn based on results obtained from a single species may easily be misleading. The measure of mRNA transcription varies in rodents. As an example, specific genes involved in cholesterol and unsaturated fatty acid biosynthesis are upregulated by specific inhibitors in hepatocytes, and the extent of inhibition was greater in rats than in mice [94].

Besides, opposing effects can be seen in specific cases. The effect of angiotensin II inhibits STC-1 gene expression in male C57Bl/6 mice; however, it has a stimulatory effect on male Wistar rats [95]. A strong difference can be found between mouse and rat gene expression in the immune system and in the case of some specific neuronal genes in the RNA-Seq atlas [96]. The disrupting effects of EDs have a robust effect on gene expression, which can be different between species. Dichlorodiphenyltrichloroethane (DDT)—a strong ED—possesses a potent estrogenic activity and thus alters gene expression in rodents. However, 51 specific genes exhibited species-specific uterine expression after DDT treatment between Sprague–Dawley rats and C57BL/6 mice [27]. A difference can also be seen in humans compared to animals, possibly rendering animal models obsolete. In the central nervous system, specific mechanisms in the regulation of rodent and human brain-derived neurotrophic factor genes differ substantially [97]. 

### 4.3. General Observations

Without EDs and E2 and/or T3, cells are in an environment that can be interpreted as a “hormone deprived” state. If both of these hormones are added to the culture medium, the experimental setup is closer to the physiological status of the neural tissue. ED treatments caused a difference between rat and mouse samples; however, this phenomenon can be seen in the untreated “ntC” samples as well. Specific receptor mRNA levels of mouse ERα and TRβ are significantly higher than in the corresponding rat samples, TRα results are higher in rats, and ERβ mRNA levels were nearly equal in both species. Results in this study were normalized to the corresponding ntC values, thus removing the influence of interspecies differences found between the basic, physiological receptor expression levels.

In all of the investigated receptors, hormone withdrawal caused different responses in mice. Here, the lack of T3 (E2 treatment groups) caused an upregulation in all of the examined receptors; however, only ERα and TRβ receptor mRNA upregulation can be seen as the effect of E2 deprivation (TH treatment groups), compared to rats (where all of the examined receptors were downregulated). This means that the basic regulation between the estrogen- and thyroid systems are different in these two species, and the difference can originate from interspecies differences between ligand preferences and relative binding affinities of the target receptors. ERs differ in this attribute due to the variability in the amino acid sequence within their respective ER ligand binding domains [98]; possibly, a similar variation can be found in TRs between species. 

The results in this paper clearly indicate that all of the EDs interfere with the physiological hormonal regulation of TRα, TRβ, ERα, and ERβ mRNA expression. Induced changes were more potent in rats than in mice; furthermore, a notable difference between treatments could be observed (compared to its respective ntC) in all of the samples from rats except after the “BPA only” treatment. The latter had a detectable but weak effect only on ERα receptor expression. In contrast, only around 50% of the experiments resulted in notable changes in mouse samples. Analyzing the results of these experiments, rat neuronal cultures seem to represent a more sensitive test model for ED effects than mouse-derived neuronal cultures. 

### 4.4. Expression of Estrogen Receptor Alpha (ERα)

In the case of the applied only hormone-treated (E2 and T3) groups, a great difference was found between the two species in ERα mRNA expression levels. In rat cerebellar cells, both hormones had a negative effect on transcription; however, in mice, both hormones increased ERα expression compared to non-treated controls. Hypothetically, this phenomenon can be a result of the traumatic nature of the tissue processing during the seeding of the cultured cells (surgery, enzymatic tissue treatment, cell harvesting, and cell culture preparation). Receptor expression of ERα is affected by developmental and brain region-specific changes during cellular and tissue development; furthermore it is heavily influenced by trauma (e.g., neuronal injury) [99,100]. The external trauma on the cerebellum can activate specific neuroprotective pathways possibly leading to notable changes in the expression pattern and expression intensity of ERα. 

The same effect can be seen in the case of BPA-only treatment; however, BPA with E2 caused an upregulation in both species. With BPA + T3, the effect disappeared in mice although remained high in rats, acting both as an agonist and antagonist of ER-α. Similar results were observed in the experiment by Hiroi et al., where higher doses of BPA (1 µM) exhibited the same actions on ERs [101].

ERα mRNA expression in mice is unaffected by ZEN, but in rats, the mycotoxin increases the mRNA levels twentyfold. Here the presence of both hormones significantly decreased ERα expression compared to ZEN, but the overall levels remained higher than what was measured in rat ntC. Similarly, increased levels can be seen in mice after hormone and ED co-treatment; however, the effect is stronger in rats than in mice. 

Arsenic only caused a notable change in rat samples but left mice granule cells unaffected in all treatment groups. It seems that mouse cells do not respond to the increased As levels with elevated mRNA expression, possibly due to differences in the signalization pathways leading to gene expression when compared to rats. The “point of divergence” is not yet known. 

### 4.5. Expression of Estrogen Receptor Beta (ERβ)

Similarly to the changes of ERα, E2 alone decreased ERβ expression in rats but increased it in mice. A negative effect on receptor expression can be observed after T3 treatment in rats; however, mice cultures were unaltered after hormone application. 

The most remarkable species-specific differences can be detected in the case of ERβ expression, in which ED-treatment effects evoked notable effects in rat but were absent in mice. Transcription levels strongly increased in every rat sample except in the BPA group (possibly due to the estrogen-agonistic BPA effect which was supported by the administered E2 or T3), making the ERβ receptor a useful indicator of ED characteristic in the future but only in rat models. BPA-induced effects were elevated by E2 and T3; on the other hand, hormone co-treatment lowered the expression changes after the ZEN application. E2 had a cumulative effect on ERβ receptor expression with As while a down-regulated receptor expression was detected if As was combined with T3.

Mouse samples were mostly unaltered by EDs (alone or in combination with hormones), except the As + T3 treatment that caused a minor downregulation. Similarly to our mice results, Davey et al. (2007) reported that As in low doses lack any significant effect on ERβ in chicken embryos, but in higher doses, As lowers transcription through the inhibition of estrogen-responsive element (ERE) expression [53,102]. Theoretically, T3 can alter gene expression through the previously mentioned cross-regulation between THs and E2, thus lowering ERβ levels after a low dose As treatment. 

### 4.6. Expression of Thyroid Hormone Receptor Alpha (TRα)

Changes of E2 treated groups show a slight receptor upregulation in mice but a minor downregulation in rats. BPA alone induced a change in rats only if the ED was combined with the natural hormones, suggesting an augmenting effect with the hormones on the target receptor. Mouse neuronal cultures exhibit a high expression of TRα after BPA application, with or without E2. The absence of estrogen but the presence of T3 dissipates this effect. Understanding the complex interplay between TR and ER regulation in the cells can possibly explain this phenomenon [34,36]. 

The effects of ZEN and As are completely different between species, both in the presence or absence of hormones. TRα receptor mRNA was significantly lower than ntC in mice; however, in rats, we have found a strong six- to ten-fold possible ligand selective upregulatory effect. This difference is the most conspicuous in our experimental data and strongly displays the diversity between rodent species (used in experiments detecting ED effects).

### 4.7. Expression of Thyroid Hormone Receptor Beta (TRβ)

E2 and T3 treatments show a reverse expression pattern in TRβ mRNA between mouse and rat neuronal cultures. In the case of the E2 only treated cultures, this pattern can be observed in the case of all investigated receptors, suggesting a major difference between rat and mouse receptor regulation in the absence of physiologic TH signals. In the case of TRβ regulation, a similar phenomenon was observed under E2 deficiency.

In groups exposed to BPA and native hormones, a strong upregulation was observed. The rate of mRNA expression was higher in rats than in mice; nevertheless, our data clearly show the modulatory effect of BPA on the TRβ receptors. Interestingly, BPA alone only caused a change in mice but not in rats.

ZEN and As has a similar effect on TRβ receptors. The result of ED treatment alone and with hormones resulted in a strong increase of receptor mRNA expression in rats; however, in mice only a minor upregulation was seen in ZEN alone and in As + T3. The pattern is similar with our data from the ERβ receptor experiment; it seems that the main effect of ZEN and As action is TRβ receptor change in our model, the mentioned EDs do not influence mouse neurons through ERβ or TRα receptors. 

## 5. Conclusions

To prove that a substance can exert an endocrine-disrupting effect, numerous other possibilities needed to be excluded from the experiment [22], some originating from the tested molecule, others from the experimental species, or the environment of the study. Improper testing can lead to false identification; a good example is styrene, a substance that was identified as an ED wrongfully due to the overlooked workplace-related stress as an environmental factor in the experiment [103]. The possibility of differences between species in response to EDs can also be a strong influencing factor in ED testing protocols.

Our hypotheses are supported by the results of this study in more ways. Significant differences were measured between rat and mouse ER and TR mRNA expression levels, but the differences in ED effect between species differed not only in the order of magnitude but also in the direction or trend of the effect. The inhibitory or stimulatory nature of EDs was not similar between species, thus supporting the idea of species-specific ED action on the cellular level. This manuscript describes the first part of a complex series of experiments. According to the results, our next step will be to determine if a difference can be found between the sensitivity of a given species. Susceptibility against endocrine disruption is profoundly affected by the concentration of EDs; thus, the next step in our research is to shed light on the species-specific response in a concentration-dependent manner.

There are numerous affected signaling pathways after ED exposure in the animals. ERs and TRs are considered conservative receptors—for instance, in the case of ERα, ligand-binding affinity shows a distinct similarity between species [104,105]—however, the cellular composition can differ between rodents. Not only the specific “route of action”—the amounts of innate enzymes or receptors and specific enzyme isoforms—can be different between species, but the overall metabolic activity of the animals or the pace of development can vary between mice and rats as well. By considering the species-specific responses in our experiments, we can decrease the risk of ED treat misidentification and provide more accurate data for the future testing and identification of EDs.

## Figures and Tables

**Table 1:** Primer sequences used for qRT-PCR analysis.

Target Gene (Mouse)	Primer Sequence 5′–3′		Reference
ERα	Forw.	GGA ACT GTC TGC CCA TCG TT	
	Rev.	GAA CCC AGG GCT GCC TTA C	
ERβ	Forw.	AAC CTT CCT CTT GGG CAT CG	
	Rev.	TTT CAT CCG GTT CTC CCA CC	
TRα	Forw.	ACC GCA AAC ACA ACAT TCC G	
	Rev.	GGG CCA GCC TCA GCT AAT AA	
TRβ	Forw.	CGA GGC CAG CTG AAA AAT GG	
	Rev.	CTC AGC ACA CTC ACC TGA AGA	
Gapdh	Forw.	TGA AAT GTG CAC GCA CCA AG	
	Rev.	GGG AAG CAG CAT TCA GGT CT	
**Target Gene (rat)**	**Primer sequence 5′–3′**		
ERα	Forw. Rev.	CAG CAG CGA GAA GGG AAA CA GGG CGG GGC TAT TCT TCT TA	[62]
ERβ	Forw. Rev.	TCC CAG CAG CAG TCA GTC CGA ACA CCG CCA CAC AAC CAC CCT	[62]
TRα	Forw. Rev.	TGG GCA AGT CAC TCT CTG C TCC TGA TCC TCA AAG ACC TC	[60]
TRβ	Forw. Rev.	AAT GTC CGA AGC CTG CCT G CAG CCT TCA CAG GTG ATG C	[61]
Beta-actin	Forw.	ATT TGG CAC CAC ACT TTC TAC AAT	
	Rev.	GTC AGG CAG CTC ATA GCT CTT CTC	
